# Supplementary material for: Photocatalytic Performance of Sol-Gel Prepared TiO2 Thin Films Annealed at Various Temperatures
Source: Materials (Basel). 2023 Aug 7;16(15):5494. doi: 10.3390/ma16155494 (PMC10419699; doi:10.3390/ma16155494)
Supplement: Supplementary file 1 [file materials-16-05494-s001.zip › materials-2508974-supplementary.pdf]

materials

MDPI

Support Information

Photocatalytic Performance of Sol-Gel Prepared TiO_2 Thin Films Annealed at Various Temperatures

Lu He [1,2,*], Dietrich R.T. Zahn [1,2], Teresa I. Madeira [1,2]

[1] Semiconductor Physics, Chemnitz University of Technology, D-09107 Chemnitz, Germany
[2] Center for Materials, Architectures, and Integration of Nanomembranes (MAIN), Chemnitz University of Technology, 09107 Chemnitz
* Correspondence: lu.he@physik.tu-chemnitz.de

Image processing protocol for the determination of the surface area of TiO_2 thin film samples

The optical images of all samples are obtained using a cell phone camera always at the same fixed position under the same light conditions. It is important to note that all the silicon substrates may have arbitrary shapes and also all samples have an "Edge" due to the spin coating process and post annealing. A typical optical image can be seen in Figure 1 left. A special "Studio" is created for taking these photos since the samples are reflective.

At first, the samples are placed inside a 2 x 2 cm square, which is manually drawn on a white paper. This square is also used to define the scale bar for the image processing. Secondly, the software ImageJ is used for extracting the "blueish" and violet region inside the pre-defined square based on the color contrast defined by the software. Finally, the masked area is transferred into a black-white binary image (see Figure 1 right) to calculate the "black" area, which corresponds to the area of TiO_2. For all samples, strictly following the steps mentioned above creates a robust determination of the total effective TiO_2 thin films areas that are used for the photocatalytic reactions.

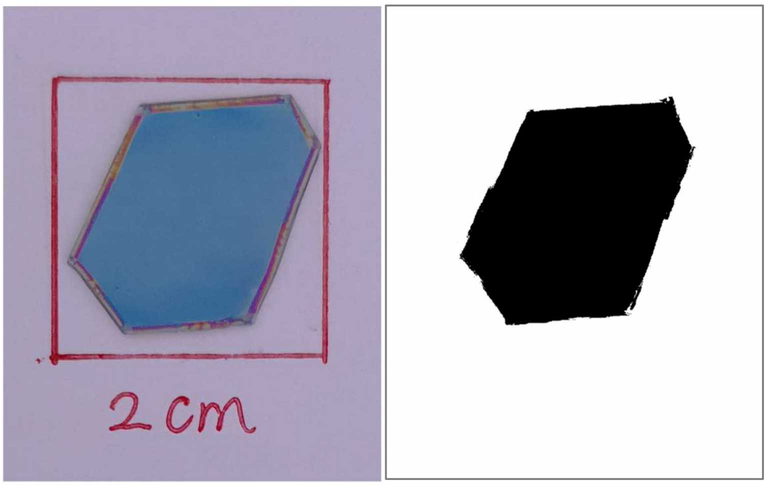

Figure S1. Left: a typical optical image of a sample; **right**: the effective area that is extracted using imageJ software based on the colour difference of the picture.
